# British Medicinal Plants

**Published:** 1891-09

**Authors:** Alfred Heath

**Affiliations:** London, England


### BRITISH MEDICINAL PLANTS.

Alfred Heath, M. D., F. L. S., London, England.

### Order 25.—Leguminosæ. (Continued.)

*Sarothamnus Scoparius* (Common Broom).—Broom tea is a very old remedy in dropsies, jaundice, etc. It acts powerfully as a diuretic; it has also been used as a purgative; it seldom fails to operate in either case; the seeds have been used as well as the top; the ashes of the burnt broom are diuretic, and they used to form an ingredient in diuretic wines. The seeds have also been roasted, and used as coffee; the bark is used for tanning.

*Ononis Spinosa* (Thorny Rest-harrow, Petty Whin, Ground Furze. Called Rest-harrow on account of the strength of its roots.)—Sheep are said to be very fond of this plant. It has been recommended as a remedy in jaundice, and for stone, suppression of urine, etc. It is said to have the power to break or dissolve stone. In the old writings it was recommended as a cure for *hernia carnosa* or fleshy rupture (sarcocele). Decoction of the powdered root in wine or spirit was taken for some months, and is said to have cured cases that were deemed incurable by medicine; it has been used in obstruction of the liver and spleen, and for indurated ulcerations.

*Melilotus officinalis* (Common Melilot).—A comparatively common English plant. The medicinal action of the Melilots is probably similiar to the following plants, as their active principle is the same, namely: Coumarine; the vernal grass *Anthoxanthum odoratum* (order Gramineæ); the woodruff *Asperula odorata* (order Rubiacea), as also Tonquin bean, *Dipterix odorata,* called also Tongo (order Leguminosæ). These plants are known to contain Coumarin. There are probably a great many others. Boiling Nitric acid converts Coumarin into Picric acid. A hot solution of Potash converts it into Coumaric acid, and eventually Salicylic acid. Preparations made from Melilot have been used successfully in treating hard tumors and inflammations in the eyes, and other parts of the body, as the rectum, uterus, etc., for spreading ulcers in the head, pains in the stomach, pains in the ears, *headaches*. Melilot *expels wind from the stomach*, reduces swelling of the spleen, *removes films from the eyes, strengthens the memory*, is said to be *effectual for sudden loss of the senses*, and *apoplexy;* applied externally the green plant relieves the pains of suppuration and causes discharge. A proving of Melilotus will be found in Hering's *Guiding Symptoms*, made in 1852, but unfortunately the proving is said to be made from a preparation of two kinds of Melilot—namely, *Melilotus Officinalis* and *Melilotus Alba*. This to my mind completely destroys its value, and for the purpose of this paper I am unable to refer to any of the symptoms produced, as I do not know whether individually they were produced by the white or yellow Melilot. I can only say that many of the symptoms *produced* by the preparation of the two plants are similar to those above mentioned, and which Melilot has the credit of having cured in times past.

*Melilotus Alba* (white flowered Melilot).—This plant is much more rare than the preceding. It does not appear to have been used in medicine, probably on account of its greater rarity. No doubt it is as good a drug, if not better than the yellow flowery plant. There is a good proving in the *Medical Advance*, Vol. XX, page 321. In the face of some of the symptoms mentioned under the previous heading which I have italicized the following symptoms given in Dr. H. C. Allen's proving are remarkable: "Head—Indolence and inability to fix the attention or comprehend the subject, rendering study extremely difficult. Total inability to study, the mind will not retain anything; even in copying, letters and words are dropped; loss of consciousness, with gushing of blood from the nose; intense frontal headache, preceded by hot flushed face. Eyes—Vision dim; a film seems to blur the sight; involuntarily rubs the eyes for relief. Stomach—Gastric discomfort; flatutent distention; eructations all day." When one remembers that these very symptoms, in the absence of any knowledge of the action of the drug on healthy persons, led to its use, and gave it a reputation in the distant past, what unprejudiced mind can fail to believe in the "law of similars"?

